# Inconsistencies in Pregnant Mothers’ Attitudes and Willingness to Donate Umbilical Cord Stem Cells: A Cross-Sectional Analysis from Saudi Arabia

**DOI:** 10.3390/jcm12093079

**Published:** 2023-04-24

**Authors:** Asma Ayyed AL-Shammary, Sehar un-Nisa Hassan

**Affiliations:** Department of Public Health, College of Public Health and Health Informatics, University of Ha’il, Ha’il 81451, Saudi Arabia

**Keywords:** umbilical cord stem cells, donation, collection, storage, pregnant, women, awareness, attitudes, behavior, registration

## Abstract

The collection and storage of umbilical cord stem cells (UCSCs) have a crucial role in improving and expanding stem cell-based therapies, which are becoming popular in Saudi Arabia and other Middle East countries. Many patients and families in Saudi Arabia depend on private cord banks in foreign countries to purchase stem cells, which has financial and medical implications. The current study aims at determining the predictors of current registration status and willingness to donate cord blood stem cells among expectant mothers in Saudi Arabia. A cross-sectional study collected data from 714 expectant mothers from all thirteen regions of Saudi Arabia in December 2022. The online survey questionnaire assessed women’s awareness, direct and indirect exposure to stem-cell therapy, sources of knowledge, willingness, reluctance, and current registration status to donate cord blood. Although women demonstrated higher acceptance and lower rejection towards the donation of UCSCs, just one percent (*n* = 7; 1%) of expectant mothers in this sample are registered with the Saudi Stem Cell Registry. Overall, 48% indicated their willingness to register in the future. Both correlational analysis and multiple regression analysis demonstrated that awareness significantly predicted willingness to donate (*p* < 0.01), and rejection attitudes were negatively related to willingness to donate (*p* < 0.001). Although the mean scores on acceptance were high, they were not found to be significantly associated with willingness to donate. Prior direct and indirect exposure to stem cell therapy appeared to be the strongest predictor of pregnant women’s willingness to register (*p* < 0.001). Findings suggest that acceptance attitudes do not have a symmetrical relationship with intention. Women’s prior exposure to stem cell therapy was the most significant factor; therefore, findings demonstrate that currently women are relying on their firsthand experience to decide about cord blood donation rather than the information obtained from other sources, such as social media and the internet. Though attitudes were not identified as significant predictors in the statistical models, awareness was a relevant factor, and the findings signify increasing awareness in various target populations to enhance the probability of intention to donate cord stem cells.

## 1. Introduction

Regenerative medicine involving stem cell transplantation has shown positive results in treating many chronic health conditions and terminal illnesses, such as type 1 diabetes, cardiac diseases, Parkinson’s disease, Alzheimer’s disease, cancer, osteoarthritis, spinal cord injuries, and burns [1]. These therapies primarily use any type of human stem cells, such as embryonic stem cells (ESCs), umbilical cord stem cells (UCSCs), and adult stem cells, for transplantation due to their regenerative ability that is required for the restoration of diseased tissues [2]. However, the underlying biological mechanisms that enable stem cells to assist in tissue and organ recovery are complex. Researchers and clinicians are thus always in search of suitable and rich sources of stem cells that have the potential to segregate into several lineages and are appropriate matches for clinical application [3].

In the stem cell hierarchy, cord blood cells possess multipotent properties. Some studies have demonstrated their pluripotent nature, which means they can differentiate themselves into a wide range of different types of cells, including neural, epithelial, endothelial, and myotubes [4,5]. Although there is ongoing research on various types of cord-blood-derived stem cells, mesenchymal stem cells extracted from the umbilical cord have shown their efficacy in treating immunodeficiency in children due to their enhanced capabilities of proliferation and differentiation [3,6]. Cord blood is also a significant source of hematopoietic stem cells that have been employed effectively in designing regenerative therapies for life-threatening diseases, such as leukemia, malignant lymphoma, anemia, cancer, and congenital heart diseases, despite some limitations [7].

Umbilical cord blood (UCB) is considered medical waste. Therefore, the storage and collection of stem cells derived from umbilical cord blood are less controversial regarding social and ethical implications than embryonic stem cells, which may involve the destruction of the embryo or fetus [8]. Moreover, umbilical cord blood collection is a relatively simple procedure that can be carried out during the delivery without any additional medical procedures. There are two key methods usually employed to collect UCB from the cord vein: before the placenta is delivered (in utero) or after the placenta is delivered (ex utero). These procedures should be applied only by adequately trained medical staff [9]. Proper documentation is important to ensure when preserving the UCB, and the collection bag should be appropriately labeled to avoid missing stem cells from one donor to another. The umbilical cord and placenta blood (UCPB) units collected should be forwarded to the processing lab, preferably within 24 hours or less. It is recommended to implement best practices during the collection in accordance with UCPB bank guidelines [10].

Cord blood banking was initiated in the early 90s and has grown faster in various world regions in the last decade [8]. Globally, there are about 700 million cord blood units, of which over 500 million are private. The global cord blood banking services market size is projected to reach $4.5 billion by 2030 [11]. According to available estimates, more than forty thousand UCB transplants have been carried out to treat various types of cancer and other diseases [12].

UCB is banked in either private or public banks. Private banks charge money for such storage and provide it to families on demand, whereas couples or women can enroll themselves to donate cord blood stem cells at public banks. At the time of delivery, UCB is collected and then stored at a public bank free of charge, and people who need it can purchase it from them. King Abdullah International Medical Research Center Cord Blood Bank (KAIMRC CBB) is authorized to function as a public bank for cord blood units in Saudi Arabia. Currently, Saudi law prohibits the operations of private UCB banks.

The key advantage of public banking is that it is “not-for-profit” and provides UCB to recipients either at minimal cost or for free, thus addressing the treatment needs of those who could not afford stem cell-based therapies at regular cost and incorporating issues related to equity and social justice [13]. Moreover, public UCB banks are significantly more ethnically diverse and can thus cater to the needs of diverse populations. However, there are some challenges, such as the increased burden on the health system and the allocation of financial resources for the maintenance of public cord blood banks. The solution to this has been generated by charging a large sum of money for the storage of UCB for personal use. Nonetheless, this solution raises questions about the moral and ethical values attached to altruistic donations of umbilical cord blood cells (UCBCs) and health equity [13,14]. There are some limitations, such as the fact that the storage of all public UCBC units does not satisfy the standards set by NetCord and the Foundation for the Accreditation of Cellular Therapy (FACT). The lack of quality control due to dysregulations in public cord blood banking limits access to good-quality specimens for transplantation [15].

The ethical considerations of collecting UCB donations involve informed consent for collection, which should begin before labor and delivery. Donor eligibility should be determined by ruling out risk factors for transmissible and genetic diseases [9]. Collection must not interfere with normal delivery care. The timing of clamping the umbilical cord after birth is also another ethical concern and is controversial due to a lack of consensus on when the umbilical cord ought to be clamped and cut because the cord is still a source of nourishment and waste removal until it is clamped [16]. Immediate reporting is needed in the case of unexpected adverse reactions during collection, and supportive counseling should be provided in the case of abnormal findings [9]. Moreover, the ethical principles of respect for human dignity and integrity, autonomy, beneficence, doing no harm, and proportionality that involve maximizing benefit at our expense should be adhered to in the collection of UCBC donations [17]. Maintaining the confidentiality of data and protecting the privacy of donors is the responsibility of all data users, who are required to respect individual rights and donors’ expectations. The Privacy Rule, in accordance with the Health Insurance Portability and Accountability Act (HIPAA), provides a set of standards to protect patients’ privacy, set limits on who can have access to health information for donors, and ensure that patients have adequate control over the use of their data. These regulations cover ‘health plans, health care clearinghouses, and health care providers that conduct financial and administrative transactions electronically’ [18] (p. 115; para 2).

Despite the growing popularity of stem cell-based treatment in various regions of the world, including the Middle East region, knowledge about umbilical cord blood donation and the use of umbilical cord stem cells in treating various diseases could be much higher. Clinical referral data indicate that many patients cannot receive hematopoietic cell transplantation without matched related donors [19]. The literature demonstrated that limited awareness about the use of UCB, the possibility of donation, and the procedures of cord blood donation are associated with a lack of intention to donate cord blood [20]. A study from the United Arab Emirates (UAE) based on a sample of 725 pregnant women demonstrated that around two-thirds of women did not have basic knowledge about umbilical cord stem cell donation and therapies [21]. This study also demonstrated that only 16% of the women in this sample were willing to donate umbilical cord blood, which increased to 91% after an educational intervention. A study from Egypt based on a convenient sample of 200 women showed that over one-third of women had average knowledge about donating UCBCs, and over two-thirds of women had negative attitudes towards this practice [22]. A quantitative study assessed the levels and nature of awareness about cord blood banking by surveying over 1100 participants from the general population in Saudi Arabia. Findings revealed that over fifty percent of respondents did not know that cord cells are sources of stem cells, the possibility of donating cord blood during delivery, UCBC retrieval procedures, or storage [23]. Another study from Saudi Arabia reported that the acceptance rate of stem cell donation in the general population was 1.8% in a sample of 506 participants. Moreover, only one-fifth had accurate knowledge about the sources of stem cells, and just over one-quarter of respondents were aware of stem cell storage and transplantation centers. For most participants, the source of information was social media or the internet [24]. The industry of cord blood banks also focuses on the marketing of private banking, and most people become aware of private banks through advertising and other marketing activities. Although India has made significant progress in private cord blood banking, women’s awareness of cord blood banking could be higher than average. In one study from India, only 26% of women in a sample of 256 expectant mothers knew about umbilical cord blood (UCB) stem cell banking, 31% were aware of private cord blood banking, and only 16% had an idea about public cord blood banking [25]. A study from Lebanon stressed the need to educate pregnant women about cord blood banking to enhance such behavior, as analysis showed that less than fifty percent were aware of the possibility of donating cord blood and its uses. Over one-quarter of those who were aware refused to donate [26]. Some prior studies from Middle Eastern countries recommended creating awareness, educating, and counseling women about stem cell donation and cord blood donation [26,27]. A study from Slovenia also reported that people from the general population were not aware of UCB banking [28]. A study of pregnant women from Italy tested the theory of planned behavior to identify the social and psychological elements of women’s decision to donate cord blood. Findings demonstrated that subjective norms and perceived behavioral control were significant determinants of intention to donate cord blood [29].

Most of the available studies from Saudi Arabia assessed the knowledge and attitudes of the general population [30], medical students [31], and healthcare providers [32] toward stem cell donation, therapy, and research. However, only some published studies directly focused on assessing expectant mothers’ awareness, attitudes, and behavior toward UCB donation. The future trends focus on the penetration of umbilical cord blood banking services in maternity hospitals, and women who are expecting to deliver babies are the primary stakeholders in deciding on donating cord blood for the retrieval of stem cells. It is, therefore, imperative to study and identify factors that may be associated with women’s willingness or refusal to donate cord blood.

The current study assesses the willingness and registration status to donate cord stem cells among pregnant women in Saudi Arabia. Moreover, the following research hypothesis was developed to assess the predictive relationship of factors, such as direct and indirect exposure to stem cell treatment, sources of information, awareness, and attitudes, in determining the women’s willingness to donate stem cells.

1. Age, education, nationality, and religious orientation significantly predict women’s intention to donate.
2. Having a first-degree relative who has undergone stem cell donation will be positively associated with women’s willingness to donate cord stem cells as compared to those who do not have such exposure.
3. Having a first-degree relative who has undergone stem cell therapy will be positively associated with women’s willingness to donate cord stem cells as compared to those who do not have such exposure.
4. Higher levels of awareness and acceptance attitudes will significantly predict current registration with the Saudi Stem Cell Registry and willingness to donate.
5. Rejection attitudes will be negatively related to the intention to donate.

The evidence and insight gained from this study will be helpful as expectant mothers are potential donors of cord blood stem cells. Improving awareness levels will protect the public from malpractices in this area of medical services. Moreover, it will identify the need for appropriate action by local health organizations to increase the registration rates for cord blood donation among women in Saudi Arabia. Such measures will ultimately enhance the research and treatment outcomes for the target population by implementing best practices in stem cell collection and storage by local banks and decreasing the population’s dependency on private foreign cord blood banks.

## 2. Materials and Methods

### 2.1. Study Design and Sample Size Estimation

This study employed a cross-sectional survey research method. The target population is composed of women expecting to deliver babies and living in Saudi Arabia. The estimated sample size was 28 respondents from each region of Saudi Arabia, calculated by choosing a population proportion of 1.8% registration rate with the Saudi Stem Registry in the general population in Saudi Arabia [24], with a confidence level of 95% and a 5% margin of error.

### 2.2. Survey Tool

A review of survey questionnaires that focused on the assessment of awareness, attitudes, and willingness to donate stem cells and stem cell-based therapies [22,23,33,34,35] was completed. Based on the review, a set of questions was developed to devise a survey tool for this study. Field experts reviewed this survey questionnaire to assess the appropriateness of items in accordance with research objectives. Both English and Arabic versions of the survey questionnaire were prepared and pretested. The pilot testing of the study questionnaire was completed before the questionnaire was administered to study participants.

The first section of the survey explained the study objectives, assessed participants on inclusion and exclusion criteria, and obtained informed consent for participation. The second section collected information about the demographic characteristics of women, including age, education, nationality, religion, and place of residence. The third part of the questionnaire included questions to assess direct and indirect exposure to stem cell therapy, sources of information about stem cell donation, current registration status with the Saudi Stem Cell Registry, and willingness to register in the future. The last part of the survey is composed of twelve items distributed into three sub-sections. The first five questions assessed awareness about cord stem cells; the following five assessed acceptance attitudes; and the last two touched on negative attitudes. Each item is rated on a 5-point Likert scale, from strongly disagree (0) to strongly agree (4). Thus, the minimum score on the awareness and acceptance scales is 0, and the maximum score is 20, whereas the minimum score on the rejection scale is 0, and the maximum is 8. The low range of scores indicates a lack of awareness, less acceptance of cord stem cell donation, and a negative attitude. The Cronbach Alpha coefficient value was α = 0.88 computed on the awareness and acceptance attitudes scales.

### 2.3. Data Collection

**Figure 1:**
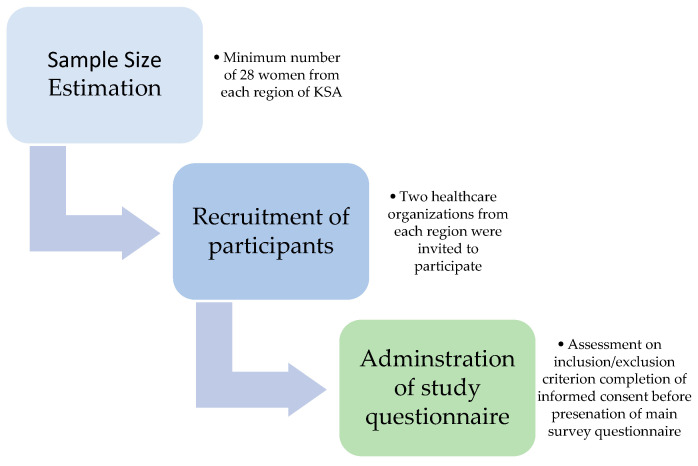
Study design and data collection process.

The data was collected from all thirteen geographical regions of Saudi Arabia, and the recruitment of a minimum number of 30 expectant mothers from each region was ensured. The data was collected on an electronic survey distributed among the target population with the help of research assistants. The participants were recruited from healthcare organizations (Figure 1). Participation in the study was voluntary, informed consent was presented at the beginning of the survey questionnaire, and respondents’ anonymity was maintained. The inclusion criteria were: (1) women aged between 19 and 45 years and expecting a baby at the time of data collection, regardless of month of pregnancy; (2) women living in Saudi Arabia; and (3) women being able to complete a self-report electronic survey questionnaire. The participation was voluntary, thus only those women who refused to participate despite meeting the criteria were excluded from the study. Data collection was completed between 26 November and 1 December 2023.

### 2.4. Ethical Approval and Considerations

The study protocol was approved by the University of Ha’il Ethics Committee (Approval Code: H-2022354) on 31 October 2022. The study was completed following the ethical codes of conduct in medical and public health research that ensure obtaining informed consent, maintaining the confidentiality and anonymity of data, and focusing on the beneficence and non-maleficence of stakeholders.

### 2.5. Data Analysis

Data were analyzed using SPSS version 25.0 software. The categorical data were reported using frequency and percentage values, and the continuous data were reported with mean scores and standard deviation. Bar graphs and pie charts are presented to display the findings. Study hypotheses were tested by applying bivariate and multivariate analysis, choosing a confidence interval (CI) of 95% and a significance level of *p* ≤ 0.05.

## 3. Results

A total of 714 women completed the online survey questionnaire. Table 1 shows the demographic background of the pregnant women who responded to this survey. The mean age of women was 29.5 years. The percentage of women between 19 and 31 years old was 58%, and between 31 and 44 years old was 42%. Around one-quarter of the women (73%) in this sample have studied up to the university level, followed by college-level education (17%) and school (10%). A large section of respondents were Saudi (89%), and a more significant proportion had a religious orientation of Muslim (96%). The study collected data from all 13 regions of Saudi Arabia. Thus, following sample size estimation, a minimum number of 30 respondents and a maximum number of 77 were recruited from each region. The proportion of women who responded to the survey was around 7% in most of the regions, with a minimum value of 5% from Al Baha and a maximum value of 11% from Mecca.

Table 2 shows the percentage of responses by women to two direct questions to assess the basic familiarity of pregnant women with stem cells or umbilical cord cells. Out of the total, 7% of women in this study were unaware of both stem cells and umbilical cord cells; 6% were unaware of stem cells, and 8% were unaware of umbilical cord cells. Women who were familiar with stem cells reported social media as the most common source of information (58%), followed by formal education (18%) and online sources (11%). Only 4% and 3% of the women reported friends and TV programs as sources of information about stem cells, respectively.

**Figure 2:**
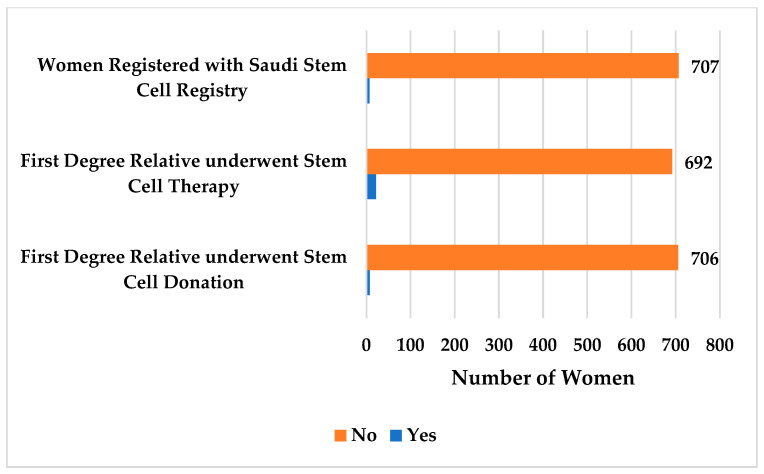
Registration with the Saudi Stem Cell Registry and exposure to stem cell donation and therapy.

Figure 2 shows that only one percent (*n* = 7; 1%) of women are currently registered with the Saudi Stem Cell Registry, and the same proportion of women (*n* = 8; 1%) reported that their first-degree relative(s) underwent stem cell donation. Nonetheless, three percent (*n* = 22; 3%) of women reported that their first-degree relative(s) underwent stem cell therapy.

The mean scores on levels of awareness, acceptance, and rejection attitudes towards umbilical cord stem cell donation and therapy are presented in Table 3. The mean score on awareness was 13.8 (S.D. = 2.73), thus indicating moderate levels of awareness. The mean score on acceptance attitudes was 17.5 (S.D. = 2.7), demonstrating higher levels of acceptance for umbilical cord stem cell donation, and the mean score on rejection attitudes was 0.44 (S.D. = 1.03), illustrating low levels of rejection attitudes towards umbilical cord stem cell donation among women in Saudi Arabia who are expecting to deliver babies.

Findings show that women reported high levels of awareness about diseases that are reliably treated by stem cell therapy (M = 3.43; S.D. = 0.67). In addition, the mean score on awareness of the potential benefits (M = 3.40; S.D. = 0.65) was higher than awareness of the potential risks (M = 2.80; S.D. = 0.94). However, means scores show that women had low levels of awareness (M = 2.89; S.D. = 0.82) about the Saudi Stem Cell Registry, the only public cord blood bank collecting and storing stem cells. Women’s awareness about other organizations that collect stem cells from donors in Saudi Arabia was also low (M = 1.37; S.D. = 0.94).

Pregnant women demonstrated higher levels of acceptance attitudes toward umbilical cord stem cell donation for treatment (M = 3.49; S.D. = 0.62) as compared to umbilical cord stem cell donation for research purposes (M = 3.23; S.D. = 0.67). In addition, women hold acceptable attitudes towards the practice of large-scale umbilical cord stem cell donation (M = 3.69; S.D. = 0.58) and favor the idea of storing umbilical cord stem cells for a future purpose (M = 3.62; S.D. = 0.65).

The women in this study demonstrated low levels of rejection attitudes, and they did not seem to believe that umbilical cord stem cells pose any threat to human life (M = 0.22; S.D. = 0.61) or should not be practiced (M = 0.22; S.D. = 0.57).

**Figure 3:**
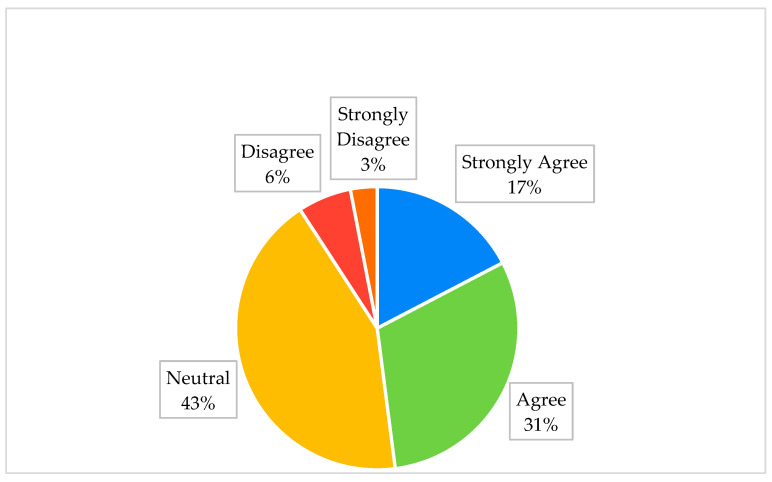
Percentage of pregnant women willing to register with the Saudi Stem Cell Registry in the future.

Figure 3 shows that only 124 (17%) of the women in this study showed a strong willingness to register with the Saudi Stem Cell Registry, and around one-third of the women (*n* = 218; 31%) agreed to register in the future with the Saudi Stem Cell Registry, while most of the women held neutral attitudes (*n* = 305; 43%). Overall, a small proportion of women (*n* = 25; 3%) strongly disagreed with registering in the future. The analysis showed that most women were aware of the need for informed consent from women for umbilical cord stem cell donation (*n* = 651; 91%).

**Figure 4:**
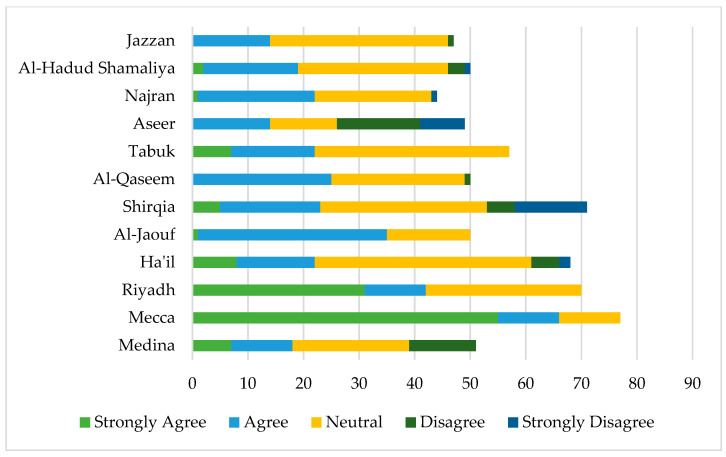
Willingness to register with the Saudi Stem Cell Registry across thirteen regions in Saudi Arabia.

Figure 4 shows the willingness of expectant mothers to donate umbilical cord stem cells from different regions in Saudi Arabia. A higher percentage of women from Mecca (44%) and Riyadh (25%) were willing to register as compared to other regions. A lower percentage of women (6%) from the Jazzan and Aseer regions demonstrated a willingness to register for donation. A higher proportion of women from Hail (12%) and Tabuk (11%), however, demonstrated a neutral response.

Table 4 presents the mean differences in pregnant women’s awareness, acceptance, and rejection attitudes towards umbilical cord stem cell donation and transplantation. Findings show there was no significant mean difference across age on awareness and acceptance; however, women in the age category of 19–30 years had significantly higher mean scores on the rejection scale (*p* < 0.05). In addition, women who studied up to the university level had significantly higher mean scores on awareness (*p* < 0.001) and acceptance (*p* < 0.01). Non-Saudi women in this study had statistically significantly higher mean scores on awareness (*p* < 0.01) and acceptance (*p* < 0.001), whereas Saudi women had higher scores on rejection attitudes (*p* < 0.001). There were non-significant mean differences in awareness levels, acceptance and rejection attitudes, and willingness to register between Muslim, Christian, and Hindu women. However, the post-hoc analysis revealed that Hindu women, in comparison to Muslim women, had a higher mean score on acceptance attitudes (*p* < 0.05) and a non-significant difference with Christian women. The study sample included pregnant women living in thirteen regions of Saudi Arabia, and results showed that women living in Shirqia had higher mean scores on awareness and acceptance and the lowest mean scores on rejection attitudes, whereas women from Al Baha had the lowest mean scores on awareness and acceptance and the highest mean score on rejection attitudes (*p* < 0.001).

Regarding the mean differences in willingness to register for donation, findings demonstrate a significant difference across nationalities. Saudi nationals had higher mean scores on willingness as compared to non-Saudi respondents (*p* < 0.05). Moreover, the differences were statistically significant across regions. Respondents from Mecca had the highest mean score (M = 3.57; S.D. = 0.73), and respondents from Aseer regions had the lowest mean score (M = 1.65; S.D. = 1.07) at *p* < 0.001.

Table 5 shows there were significant mean differences in awareness, acceptance, and rejection scores across categories of sources of knowledge. Women who had formal education had significantly higher mean scores both on awareness and acceptance (*p* < 0.001), whereas women who used social media as sources of information had a significantly higher mean score on rejection attitudes (*p* < 0.001). Direct and indirect exposure to stem cell donation and treatment was related to awareness and acceptance (*p* < 0.001).

Findings show that participants who reported that their source of knowledge about stem cells was TV programs had higher mean scores on willingness to register, with statistical significance at *p* < 0.01. In addition, women who reported having a first-degree relative who underwent stem cell donation or stem cell therapy had a higher willingness to register, with statistical significance at *p* < 0.01 and *p* < 0.001, respectively.

Table 6 shows that awareness was strongly positively associated with acceptance (r = 0.75; *p* < 0.001) and weakly but significantly associated with willingness to register with the Saudi Stem Cell Registry (r = 0.01; *p* < 0.01), whereas it was moderately negatively associated with rejection attitudes (r = 0.37; *p* < 0.01). Acceptance was significantly and strongly negatively associated with rejection attitudes (r = 0.62; *p* < 0.01), but demonstrated a non-significant association with willingness to register. Rejection attitude scores were weak but significantly negatively associated with willingness to register (r = −0.85; *p* < 0.05).

Table 7 shows that in multiple regression analysis, nationality as Saudi (*p* < 0.01), formal education as a source of information for stem cell donation and therapy (*p* < 0.01), direct and indirect exposure to stem cell therapy (*p* < 0.001), and awareness about umbilical cord stem cells (*p* < 0.05) significantly positively predicted willingness to register with the Saudi Stem Cell Registry. Conversely, rejection attitudes significantly negatively predicted willingness to register (*p* < 0.001). Among all the results, direct and indirect exposure to stem cell therapy appeared as the strongest positive predictor of willingness to donate (β = 0.19; 95% CI = 0.64–1.48; *p* < 0.001) and rejection attitudes appeared as the strongest negative predictor of willingness to donate (β = −0.17; 95% CI = −0.24–−0.07; *p* < 0.001).

## 4. Discussion

This study, conducted on a sample of 714 expectant mothers living in Saudi Arabia, aimed to assess the women’s current registration status to donate umbilical cord stem cells and willingness to register with the Saudi Stem Cell Registry in the future, as well as its social and psychological predictors. Overall, the study has several strengths, as it is based on a large sample of women expecting to deliver and potential candidates to donate cord blood. In addition, the study participants were recruited from all regions in Saudi Arabia; thus, the generalization of findings is high and applicable to the national and regional levels. Moreover, the study explored some of the critical social and psychological variables, such as women’s prior exposures, knowledge, sources of knowledge, and acceptance and rejection attitudes, as a determinant of intent to donate. Considering that previous studies demonstrated that registration rates with the Saudi Stem Cell Registry in the general population are less than one percent, it is essential to explore its predictors.

Findings from this study showed that around 90% of women admitted having a basic familiarity with or idea about stem cells and umbilical cord stem cells. However, on the awareness scale that assessed some specific aspects of awareness, including knowledge about types of disease treated by stem-cell therapy, the potential benefits and risks of stem cell therapy, and awareness about public and private banks for donation and storage, women demonstrated low to moderate levels of awareness. Most of the women reported that social media was the most common source of information. These findings suggest that pregnant women’s subjective assessment of their current levels of awareness about umbilical cord stem cells cannot be ascertained because most of the women reported that their information sources are social media and the authenticity of the information shared through social media is relatively low. Current study findings align with previous studies from Saudi Arabia that assessed the awareness about stem cells in the general population and reported that, despite over two-thirds of participants having familiarity with the concept of stem cells, less than twenty percent had correct responses on questions that asked about sources of stem cells, the efficacy of stem cell-based therapies, and stem cell banks [24]. Even an earlier study that mainly determined the level of awareness of umbilical cord blood banking also indicated a lack of awareness about cord blood donation and its uses in stem cell therapy and research among the general population in Saudi Arabia [23]. Based upon these observations, it can be inferred that despite increased access to information about stem cells through social media in the past few years, which has been the most common source of information reported by participants, the overall levels of accurate knowledge about stem cell donation, therapy, research, and donor registration agencies are low among pregnant women in Saudi Arabia. This is further validated by the fact that only 1% of expectant mothers in this study sample are currently registered with the Saudi Stem Cell Registry for donation, 1% reported that their first-degree relative donated stem cells, and 3% reported that their first-degree relative underwent stem cell therapy.

Additionally, women reported greater awareness of potential benefits as compared to risks, and this is possibly due to the fact that social media campaigns and marketing of cord blood banking portray the beneficial outcomes of such therapies and undermine the potential risks associated with stem cell-based therapies. However, women had low levels of awareness about the Saudi Stem Cell Registry, which is more likely the underlying reason for the low levels of registration, which are only 1% among women and align with previous research [24]. There are no other private stem cell banks in KSA, and women also demonstrated low levels of awareness about these banks.

Overall, there is higher acceptance for stem cell donation, though the overall rate of current registration with the Saudi Stem Cell Registry is meager. This could be due to a lack of awareness about registering or to a disconnect between acceptance and its actual translation into behavior. The social desirability factor could be a possible explanation for this higher score on acceptance, and this has been supported by the previous literature, where despite the high rate of accepting stem cell donations and therapy among people in Saudi Arabia, there were still some who were reluctant to register themselves as donors [36].

Out study findings also show that most women are undecisive about whether to register for the Saudi Stem Cell Registry, and only 17% strongly agree to register. Although it is a positive sign that around 50% of women showed willingness to register, how many of them will do so is doubtful since, although a large number of people show acceptance and willingness to adopt healthy behaviors, a limited number transform those intentions into action. In a recent study of Brazilian women, only 13% of women expressed their intent to donate or store cord blood stem cells [37]. Overall, the findings depict that despite higher acceptance scores, 50% of women did not demonstrate their willingness to register with the Saudi Stem Cell Registry to donate cord blood stem cells. These findings are further validated in bivariate and multivariate analyses, where acceptance did not appear as a significant determinant of willingness to register for a donation of cord blood to banks in Saudi Arabia. Therefore, these findings suggest that practical solutions might be needed to improve the low rate of registration for stem cell donation in Saudi Arabia. For instance, information should be provided to women and their families either by their family physicians or healthcare staff who are responsible for providing maternal care. This might be beneficial to assess the eligibility of women to donate UCBCs during their routine visits and improve the rates of successful donations. Previous research from African regions has also demonstrated that health workers who deliver prenatal and obstetric care have primary roles in promoting cord blood donation [37]. Based on the findings of our recent study that assessed the knowledge and attitudes of medical professionals, we found that female professionals, Saudi nationals, and those who do not have prior work experience in stem-cell donation, therapy, or research had low levels of knowledge, less sensitivity, and a less accepting attitude [38]. Thus, overall, there is a need for education and awareness campaigns at multiple levels to expand UCB donations in Saudi Arabia.

A prior study from Italy tested the application of the theory of planned behavior to demonstrate the contributing role of attitudes in determining the intention to donate stem cells [29]. This study demonstrated that subjective norms and perceived behavioral control may influence the intention to donate stem cells for pregnant women. The current study is limited in that it did not explore subjective norms; however, the low intention to donate stem cells and the limited number of women who are currently registered with the Saudi Stem Cell Registry are explainable. Considering the social and cultural context of Saudi Arabia, where despite increased interest in stem cell-based therapies among the general population and acceptance attitudes, subjective norms may influence couples’ decisions to donate cord blood stem cells. Previous research has also demonstrated that shared decision making in collectivist societies may influence women’s intention to donate cord blood [38].

Debates related to stem cell donation and research have included the perspective of religions. In our study, a comparison of mean scores did not reveal statistically significant differences in awareness, attitudes, and willingness to donate among women from Muslim, Christian, and Hindu religions. Nonetheless, additional analysis revealed that Hindu women have slightly higher acceptance attitudes when compared to Muslim women and non-significant differences with Christian women. Our findings are comparable to a previous study that examined public opinion towards stem cell research by collecting data from Europe, Canada, and the US and demonstrated that, despite a sizeable fraction of individuals from all religions rejecting stem cell-based research, several equally religious people have been supporting it [39]. This study demonstrated that religious and moral convictions were influential in the US, whereas ‘the perceived benefit to society’ appeared as a dominant notion in Europe, and both carried equal weight in Canada [39]. Our study findings showed a non-significant difference in willingness to donate among women across all three religious orientations. These findings are understandable considering the previous literature, which was based upon the analysis of experts’ commentaries from six religions, including Islam, Hinduism, and Christianity, and concluded that none of the religions placed particular significance on or placed restrictions on umbilical cord blood banking [40].

Our study findings showed that a higher proportion of women from Mecca and Riyadh regions reported willingness to register for cord blood donation. The possible explanation could be higher levels of awareness and the fact that these regions are more populated with people from diverse social backgrounds. The multivariate findings demonstrate that prior direct or indirect exposure to stem cell therapy significantly predicts intention to register. This is understandable, as direct and indirect exposure is likely to increase awareness and understanding about the importance and use of cord blood stem cells, thus positively influencing women’s intent to donate. The analysis of mean differences supports this inference and demonstrates that women who underwent stem cell therapy or had a first-degree cousin who did had significantly higher mean scores on awareness and acceptance of stem cells.

Contrary to previous study findings that demonstrated that a positive attitude towards donation significantly predicts intent to donate [29,41], our study did not support this relationship, and it seems that, despite having positive attitudes, other factors may influence women’s intent to donate stem cells. The rates of stem cell donation are restricted to 2.2%, despite overall acceptance of stem cell research and therapy among populations in these countries. The psychological literature has also demonstrated that the relationship between attitudes and behavior is not symmetrical and that several social, individual, and collective experiences are likely to determine the adoption of healthy behaviors. These could be subjective norms or cultural norms other than levels of awareness and sources of information. Although the current study did not explore other factors such as perceived behavioral control and subjective norms or the impact of education or counseling about cord blood donation by their healthcare providers on their intention to donate, the previous literature has demonstrated the positive impact of educational intervention in healthcare settings on the intention to donate, which significantly increased post-intervention [27]. In addition, limited knowledge and awareness are essential variables in stem cell donation and treatment studies from developed countries [37].

The current study findings need to be interpreted keeping in mind the study limitations, as the analyses are based upon self-reported data. Therefore, the social desirability factor might have influenced acceptance and rejection scores. Keeping in mind that the women in the study had moderate levels of awareness and a highly positive attitude, and most women also believed that stem cell transplantation should be practiced on a large scale, less than one-quarter of women were willing to register, and only one percent have current registration. Future research should focus on developing an in-depth understanding through qualitative research to explore underlying factors that may hinder women’s or couples’ decisions to donate umbilical cord blood cells. Identifying the specific nature of apprehensions and hinderances may be helpful to improve the content and focus of educational and other interventions that aim at increasing knowledge and awareness levels, attitudes, and modifications of behaviors to promote umbilical cord stem cell donation among expecting mothers. Moreover, the current study assessed willingness but did not collect any data to determine eligibility, so future research could also include variables such as current or previous history regarding pregnancy, week of gestation, history of any abortion, or anomalous babies in the previous years. This information will be useful to determine the factors that may be associated with the successful collection of UCBCs.

## 5. Conclusions

In conclusion, the present study’s findings provide helpful insight about expectant mothers’ levels of awareness, attitudes, intent, and behaviors towards cord blood stem cell donation. Only promoting attitudes may not directly influence the women’s intentions and behavior, as this relationship is likely mediated or moderated by other social and psychological variables. Women’s prior exposure to stem cell therapy was the most significant factor; therefore, findings demonstrate that currently women are relying on their firsthand experience to decide about cord blood donation rather than the information obtained from other sources such as social media and the internet. Though attitudes were not identified as significant predictors in the statistical models, awareness was a relevant factor, and the findings signify increasing awareness in various population sub-groups. Future health education for pregnant women should include this information as an essential component of increasing cord blood donation among expectant mothers in Saudi Arabia.

## Figures and Tables

**Table 1:** Background characteristics of pregnant women who participated in the survey (*n* = 714).

Variables	Categories	Frequency	Percentage
Age (M = 29.5; S.D. = 5.2; Range = 19–44)	19–30 years	413	57%
	31–44 years	301	43%
Education	School	68	10%
	College	126	17%
	University	520	73%
Nationality	Saudi	637	89%
	Non-Saudi	77	11%
Religious Orientation	Islam	684	96%
	Hindu	16	2.0%
	Christian	14	2.0%
Place of Residence	Medina	51	7.0%
	Mecca	77	11%
	Riyadh	70	10%
	Ha’il	68	8.0%
	Al-Jaouf	50	7.0%
	Shirqia	71	10%
	Al-Qaseem	50	7.0%
	Tabuk	57	8.0%
	Aseer	49	7.0%
	Najran	44	6.0%
	Al-Hadud Shamaliya	50	7.0%
	Jazzan	47	7.0%
	Al-Baha	30	5.0%

**Table 2:** Self-assessment of knowledge about stem cells and sources of knowledge (*n* = 714).

Do You Know What Stem Cells Are?	Frequency	Percentage
Yes	672	94%
No	42	6.0%
Do you know what umbilical cord cells are?	Frequency	Percentage
Yes	657	92%
No	57	8.0%
Not aware of stem cells/umbilical cord cells	47	7.0%
Sources of knowledge about stem cells	Frequency	Percentage
Formal education	127	18%
Social Media	418	58%
Online Sources	77	11%
Friends	26	4.0%
TV Programs	19	3.0%

**Table 3:** Awareness, acceptance, and rejection attitudes towards umbilical cord stem cell donation and treatment among pregnant women in Saudi Arabia (*n* = 714).

Awareness of Umbilical Cord Stem Cell Donation and Therapy	Mean (S.D.)
Aware of diseases that are currently being treated in reliable ways.	3.43 (0.67)
Aware of the potential benefits.	3.40 (0.65)
Aware of the potential risks.	2.80 (0.94)
Aware that the Saudi Stem Cell Donor Registry is the only authorized body in Saudi Arabia to collect stem cells from donors.	2.89 (0.82)
Aware of other organizations that collect stem cells from donors in Saudi Arabia.	1.37 (0.94)
Total Awareness Mean Score	13.88 (2.73)
Acceptance Attitudes	Mean (S.D.)
It is okay to donate umbilical cord stem cells.	3.47 (0.65)
Women about to deliver should volunteer to donate umbilical cord stem cells for treatment.	3.49 (0.62)
Women who are about to deliver should volunteer to donate umbilical cord stem cells for research.	3.23 (0.67)
Stem cell transplantation should be practiced on a large scale.	3.69 (0.58)
Support allowing pregnant mothers to store cord blood stem cells for future purposes.	3.62 (0.65)
Total Acceptance Attitude Mean Score	17.51 (2.74)
Rejection Attitudes	Mean (S.D.)
Umbilical cord stem cell transplantation may open the door to the destruction of innocent human life.	0.22 (0.61)
Umbilical cord stem cell transplantation values individual lives over others and should not be practiced.	0.22 (0.57)
Total Rejection Attitude Mean Score	0.44 (1.03)

**Table 4:** Mean differences across demographic variables in awareness, acceptance, and rejection towards umbilical cord stem cell donation and transplantation (*n* = 714).

Variable	Categories	Awareness	Acceptance	Rejection	Willingness to Register
		M (S.D)	M (S.D)	M (S.D)	M (S.D)
Age	19–30 years	13.89 (2.76)	17.37 (2.84)	0.51 (1.16)	2.51 (0.97)
	31–44 years	13.88 (2.71)	17.69 (2.59)	0.34 (0.91)	2.54 (0.98)
	*t*-test	t = 0.56 (ns)	t = 1.54 (ns)	t = 2.29 *	t = 0.49 (ns)
Education	School	12.22 (2.64)	15.21 (3.84)	1.38 (1.81)	2.46 (0.76)
	College	13.22 (2.39)	17.17 (2.50)	0.56 (1.06)	2.51 (0.93)
	University	14.27 (2.71)	17.89 (2.46)	0.29 (0.81)	2.54 (0.99)
	F-test	F = 22.94 ***	F = 32.56 **	F = 38.11 (ns)	F = 0.23 (ns)
Nationality	Saudi	13.77 (2.74)	17.35 (2.72)	0.47 (1.07)	2.56 (0.97)
	Non-Saudi	14.81 (2.53)	18.83 (2.51)	0.16 (0.60)	2.26 (0.83)
	*t*-test	t = 3.14 **	t = 4.55 ***	t = 3.91 ***	t = 2.88 *
Religious Orientation	Islam	13.85 (2.74)	17.45 (2.74)	0.45 (1.05)	2.53 (0.97)
	Hindu	15.00 (1.86)	18.94 (1.73)	0.13 (0.50)	2.38 (0.95)
	Christian	14.57 (1.82)	18.00 (2.77)	0.00 (0.00)	2.93 (0.91)
	F-test	F = 1.84 (ns)	F = 2.49 (ns)	F = 2.05 (ns)	F = 1.43 (ns)
Place of Residence	Medina	14.88 (1.39)	19.06 (1.17)	0.33 (0.55)	2.25 (0.97)
	Mecca	12.27 (3.23)	16.18 (2.61)	0.18 (0.80)	3.57 (0.73)
	Riyadh	15.01 (3.03)	16.63 (3.02)	0.34 (1.12)	3.04 (0.92)
	Ha’il	14.31 (3.24)	18.01 (2.85)	0.18 (0.66)	2.31(0.88)
	Al-Jaouf	13.38 (2.42)	17.26 (2.92)	0.66 (1.20)	2.72 (0.49)
	Shirqia	15.89 (1.98)	19.42 (1.75)	0.11 (0.66)	1.96 (1.16)
	Al-Qaseem	13.52 (2.30)	17.64 (2.63)	0.62 (1.44)	2.48 (0.54)
	Tabuk	13.81 (2.31)	17.84 (2.31)	0.25 (0.78)	2.51 (0.71)
	Aseer	14.65 (2.81)	17.82 (2.84)	0.20 (0.67)	1.65 (1.07)
	Najran	13.59 (1.96)	17.23 (2.64)	0.91 (1.27)	2.48 (0.66)
	Al-Hadud Shamaliya	13.54 (2.31)	17.46 (2.66)	0.56 (1.21)	2.32 (0.74)
	Jazzan	12.47 (1.55)	16.72 (2.54)	0.64 (1.16)	2.28 (0.49)
	Al-Baha	11.53 (1.94)	15.37 (2.76)	1.77 (1.01)	2.90 (0.75)
	F-test	F = 12.73 ***	F = 9.86 ***	F = 7.99 ***	F = 22.14 ***

*p*-value significance: *** *p* < 0.001; ** *p* < 0.01; * *p* < 0.05.

**Table 5:** Mean differences in awareness, acceptance, and rejection towards umbilical cord stem cell donation and transplantation across the categories of sources of knowledge and prior exposure (*n* = 714).

Variables	Categories	Awareness	Acceptance	Rejection	Willingness to Register
		**M (S.D)**	**M (S.D)**	**M (S.D)**	**M (S.D)**
Sources of knowledge	Formal education	16.92 (2.01)	19.77 (0.82)	0.09 (0.43)	2.65 (1.03)
	Social Media	13.45 (2.14)	17.66 (1.87)	0.30 (0.73)	2.57 (0.88)
	Online Sources	13.90 (2.41)	17.23 (2.41)	0.26 (0.65)	2.29 (1.11)
	Friends	14.27 (2.25)	18.12 (1.58)	0.08 (0.27)	1.77 (1.17)
	TV Programs	13.32 (2.58)	17.05 (2.41)	0.00 (0.00)	3.42 (0.83)
	F-test	F = 63.51 ***	F = 38.84 ***	F = 3.83 **	F = 10.21 **
You or first-degree relatives underwent stem cell donation	Yes	16.75 (2.96)	18.75 (1.83)	0.25 (0.46)	3.50 (0.75)
	No	13.85 (2.71)	17.49 (2.75)	0.44 (1.04)	2.51 (0.95)
	*t*-test	t = 2.99 *	t = 1.29 (ns)	t = 0.51 (ns)	t = 2.91 **
You or first-degree relatives underwent stem cell therapy	Yes	16.82 (3.04)	19.23 (1.87)	0.23 (0.86)	3.68 (0.13)
	No	13.79 (2.67)	17.45 (2.75)	0.45 (1.04)	2.49 (0.36)
	*t*-test	t = 5.20 ***	t = 4.29 ***	t = 0.97 (ns)	8.38 ***

*p*-value significance: *** *p* < 0.001; ** *p* < 0.01; * *p* < 0.05.

**Table 6:** Correlation between awareness, acceptance, rejection, and willingness to register with the Saudi Stem Cell Registry for a donation of cord blood stem cells (*n* = 714).

	Awareness	Acceptance Attitude	Rejection Attitude	Willingness to Register
Awareness	-	0.750 **	−0.373 **	0.107 **
Acceptance	0.750 **	-	−0.629 **	0.015
Rejection	−0.373 **	−0.629 **	-	−0.085 *

*p*-value significance: ** *p* < 0.01; * *p* < 0.05.

**Table 7:** Multiple regression analysis for predictors of willingness to register with the Saudi Stem Cell Registry (*n* = 714).

	Unstandardized Coefficients		Standardized Coefficients	*t*	95.0% Confidence Interval for β	
	B	Std. Error	β		Lower Bound	Upper Bound
(Constant)	4.47	0.563		7.95	3.372	5.583
Age (Ref Category 31–45 years old)	0.020	0.041	0.018	0.48 (ns)	−0.061	0.101
Education (Ref Category University Education)	0.018	0.083	0.008	0.21 (ns)	−0.144	0.180
Nationality (Ref Category Saudi)	0.547	0.160	0.176	3.42 **	0.860	0.233
Religion (Ref Category Islam)	0.165	0.215	0.034	0.76 (ns)	−0.258	0.588
Sources of Information (Ref Category Formal Education)	0.371	0.128	0.148	2.90 **	0.623	0.120
Prior Direct/indirect Exposure to Stem Cell Donation	0.445	0.346	0.049	1.28 (ns)	−0.233	1.124
Prior Direct/indirect Exposure to Stem Cell Therapy	1.065	0.215	0.191	4.95 ***	0.643	1.486
Awareness	0.044	0.022	0.124	1.96 *	0.000	0.087
Acceptance Attitudes	−0.085	−0.023	−0.243	−3.64	−0.131	−0.039
Rejection Attitudes	−0.158	0.044	−0.170	−3.57 ***	−0.245	−0.071

*p*-value significance: *** *p* < 0.001; ** *p* < 0.01; * *p* < 0.05.

## Data Availability

The data presented in this study are available on request from the corresponding author with a reasonable reason.
